# The provision of bereavement care by general practitioners: data from a sentinel network

**DOI:** 10.1186/s12875-024-02625-9

**Published:** 2024-10-23

**Authors:** Sophie C. Renckens, H. Roeline Pasman, Nienke J. Veldhuijzen, Bregje D. Onwuteaka-Philipsen

**Affiliations:** 1 Department of Public and Occupational Health, Amsterdam UMC, location VU Medical Center, Amsterdam, The Netherlands; 2 Expertise Center for Palliative Care Amsterdam UMC, Amsterdam, The Netherlands; 3 Nivel, Utrecht, the Netherlands

**Keywords:** Aftercare, Primary care, Palliative care

## Abstract

**Background:**

Limited information exists regarding the prevalence of bereavement care provision by general practitioners (GPs) and in what cases they provide this. Insights into the current practice of bereavement care provision by GPs can highlight areas for improvement of the bereavement care practice. Therefore, we examined in how many cases GPs contacted relatives regarding bereavement care, and which case-specific characteristics are associated.

**Methods:**

This study had a retrospective cross-sectional design and used data from a clustered sample of 52 GP-practices in the Netherlands. Patient cases were included if they were one year or older and died between January 1st, 2018 and December 31st, 2022. The main outcome was whether the GP had had contact with relatives regarding bereavement care or planned to do so. Descriptive statistics were used, as well as logistic regression analyses with generalized estimating equations.

**Results:**

Following 86.4% of deaths, GPs either had contact with or planned to have contact with relatives of their deceased patients regarding bereavement care. This likelihood was higher in non-sudden deaths compared to sudden deaths (odds ratio [OR] 1.60). In cases of non-sudden death, GPs were more likely to provide bereavement care if an informal caregiver was involved (OR 3.81), or if the GP was part of a palliative care at home group (PaTz) (OR 2.78).

**Conclusions:**

In the majority of cases GPs reach out to the relatives of their deceased patients to offer bereavement care. Given their familiarity with the deceased person, particularly instances of non-sudden death, the GP seems to be well-positioned to provide bereavement care, especially support that focuses on reviewing the period leading up to the death.

**Supplementary Information:**

The online version contains supplementary material available at 10.1186/s12875-024-02625-9.

## Introduction

Considering the profound impact of losing a loved one, bereaved individuals may need bereavement care. General practitioners (GPs) seem well-positioned to provide bereavement care [1], but their current practice of bereavement care provision has been relatively little studied. Two literature reviews of somewhat older studies showed that bereavement care by GPs includes common elements such as expressing their condolences, offering advice about the grieving process, encouraging bereaved individuals to share their feelings, exploring spiritual concerns and discussing available local services [1, 2]. Often this is a one-time contact, with further follow-up initiated by the bereaved individuals themselves. However, some healthcare professionals may reach out more than once, particularly to those they perceive as coping poorly or being socially isolated [2]. Most GPs recognize bereavement care as an important aspect of their role [2]. GPs can offer support, including psychological and grief support, and facilitate access to additional services, such as referral to mental health professionals [3]. In the Netherlands, there is a guideline on grief in the palliative phase, which also entails information on care for bereaved relatives after the death of a loved one [4]. The recommendations in the guideline are in line with the elements of bereavement care found in the literature reviews as aforementioned [1, 2]. The guideline recommends healthcare professionals to have low-threshold contact with bereaved relatives at three months after the death to monitor if additional care is needed, and another conversation after six months if the development of a grief disorder is suspected [4].

The way bereaved individuals experience and cope with loss varies greatly. While many people are able to cope with bereavement with minimal support, some experience prolonged and intense grief, known as prolonged grief disorder, necessitating additional support [5]. Estimates of prolonged grief disorder prevalence vary widely, ranging from 9.8 to 49% of bereaved individuals [6, 7]. Various risk factors have been identified to contribute to the development of complicated grief, including being the partner or parent of the deceased, high levels of pre-death marital dependency and limited social support. Additionally, the suddenness of death significantly impacts grief experiences [8]. A sudden and unexpected death is associated with an elevated risk of prolonged grief disorder [9]. Relatives facing sudden and unexpected loss may have distinct needs regarding bereavement care.

Studies on bereavement care by GPs typically focus on the GPs of the bereaved individuals [10, 11], who are not necessarily the same GP as the deceased person’s GP. However, the deceased person’s GP may also play a valuable role in providing bereavement care for relatives, such as through a follow-up conversation. In palliative care, it falls within a GP’s role to care for both the patient and their relatives [12]. Moreover, bereaved individuals may seek answers to questions regarding the disease process, e.g. the end-of-life phase [10], which may be more effectively addressed by the deceased person’s physician who was most involved in that process rather than by the relatives’ own GP.

Despite several studies on bereavement care by GPs (e.g [2, 3, 10, 13]). , there remains little understanding of how often GPs offer this care to bereaved people and whether this is associated with specific case characteristics. This knowledge gap was also identified as one of the research priorities in the field of bereavement care by Ghesquiere et al. [1], which has to our knowledge not been answered since then. Insights into the current practice of bereavement care provision by GPs can highlight areas for improvement of the bereavement care practice. Therefore, we aim to investigate in how many cases GPs provide bereavement care to relatives of their deceased patients and to examine whether this is related to certain case characteristics. Consequently, the research questions are:

1. In how many cases have general practitioners had contact with bereaved relatives of their deceased patients regarding bereavement care?
2. To what extent is the provision of bereavement care by general practitioners associated with the death being expected and non-sudden?
3. In cases of expected and non-sudden deaths, what characteristics of the deceased patient are associated with the provision of bereavement care by the patient’s general practitioner?

## Methods

### Study design and data collection

This study had a retrospective cross-sectional design and was based on data from a clustered sample of 52 GP-practices in the Netherlands (the so-called Nivel Sentinel Practices network) [14]. The practices constitute a nationally representative sample of Dutch general practices and the database includes recorded data from healthcare providers. For the purposes of the current study data from 2018 to 2022 were used. For every patient that died, GPs were asked to fill in a standardised (online) death registration questionnaire, which they received immediately following the registration of the death in the clinical information system.

### Study population

Patients were included in this study if they were 1 year or older and died between January 1st, 2018 and December 31st, 2022. Patients were excluded if their main place of residence in the last year of life was a nursing home (since they received care from an elderly care physician instead of a GP), ‘other’ or ‘unknown’ or if the GP had a missing on the variable on bereavement care. People who mainly resided in a residential home during their last year of life were included because they were cared for by the GP.

### Measurements

The dependent variable is whether GPs had had contact or had plans to have contact with relatives of their deceased patient with regard to bereavement care. Answer possibilities were ‘yes, once’, ‘yes, multiple times’, ‘no, but planned’ and ‘no, and not planned’. For the main analyses, we dichotomised this variable into ‘yes (planned)’ (yes, once; yes, multiple times; no, but planned) and ‘no’ (no, and not planned). The answer category ‘no, but planned’ was included in the ‘yes’ category since in some cases the death registration questionnaire may have been completed shortly after death, leaving little time to already have had contact with bereaved relatives. Contacting relatives about bereavement care, whether already done or planned, is considered a form of bereavement care provision. Therefore, this study talks about the provision of bereavement care.

Independent variables included variables on demographics, hospitalisation and emergency unit admission, evaluation of the dying phase, palliative care and informal caregivers. Measurements are described extensively in Additional file 1.

### Statistical analysis

Statistical analyses were performed using SPSS IBM V.28. Characteristics of the deceased patient were assessed using descriptive statistics. The item on bereavement care was also analysed using descriptive statistics, in which results were stratified by the suddenness of death (sudden vs. non-sudden death). The item on bereavement care was dichotomised for further analysis. Univariable logistic regression analyses with Generalized Estimating Equations (GEE) were performed to examine whether the provision of bereavement care was associated with the suddenness of death. Among the group of patients whose death was non-sudden we explored which characteristics of the deceased patients were associated with the provision of bereavement care using multivariable logistic regression analyses with GEE. The GEE accounted for possible clustering of patient data on GP-practice level. Therefore, Sentinel practices were included as random effects. Analyses were corrected for age, gender, and year of death, by including these variables as fixed effects in the model. All variables that had a *p*-value less than 0.10 in univariable logistic regression analyses were included in multivariable logistic regression analysis, in which a *p*-value < 0.05 was considered statistically significant.

As sensitivity analyses we conducted the logistic regression analyses with GEE, in which the “no, but planned” category was included in the “no bereavement care” category instead of in the “yes bereavement care" category.

### Ethical approval

This study does not fall within the scope of the Medical Research Involving Human Subjects Act and therefore does not require ethical approval. General practices that participate in Nivel Primary Care Database are contractually obliged to: (1) inform their patients about their participation in Nivel Primary Care Database, and (2) to inform patients about the option to opt-out if patients object to inclusion of their data in the database. Dutch law allows the use of electronic health records data for research purposes under certain conditions. According to Dutch legislation, and under certain conditions, neither obtaining informed consent nor approval by a medical ethics committee is obligatory for this kind of observational studies (Dutch Civil Law (BW), Article 7:458; http://www.dutchcivillaw.com/civilcodebook077.htm, Medical Research Involving Human Subject Act (WMO); http://www.ccmo.nl/en/nonwmoresearch), and General Data Protection Regulation (AVG).

## Results

### Patient characteristics

GPs completed death registration questionnaires for 2010 patients between 2018 and 2022, of which 1779 met the inclusion criteria (225 patients excluded because the main place of residence in the last year of life was a nursing home, other place, or unknown, and six because of a missing value on the bereavement care variable). Most deceased patients were aged between 65 and 84 years (55.4%) or 85 years and older (27.0%) (Table 1). Of all patients, 57.5% were male and 42.5% were female. The most common cause of death was cancer (41.1%), followed by cardiovascular diseases (21.0%) and old age (10.6%). A total of 12.8% of patients were diagnosed with dementia: 5.9% with severe dementia and 6.9% with mild dementia. For the majority of patients the main place of residence in the last year of life was at home/with family (95.2%). The most common place of death was also at home/with family (60.1%), followed by the hospital (23.3%) and palliative care units/hospices (7.2%).

**Table 1:** Patient characteristics (*n* = 1779)

		*N*(%)
Age		
< 65 years		311 (17.5)
65–84 years		984 (55.4)
≥ 85 years		480 (27.0)
Gender (female)		756 (42.5)
Sudden death		
Yes		616 (34.6)
No		1163 (65.4)
Cause of death		
Cancer		718 (41.1)
Cardiovascular diseases		367 (21.0)
Respiratory diseases		141 (8.1)
Disorders of the nervous system		47 (2.7)
Stroke (CVA)		81 (4.6)
Old age		186 (10.6)
Other		208 (11.9)
Dementia diagnosis		226 (12.7)
Yes		
Severe		104 (5.9)
Mild		122 (6.9)
No		1536 (87.2)
Main place of residence in the last year of life		
Home/with family		1693 (95.2)
Residential home		86 (4.8)
Place of death		
Home/with family		1069 (60.1)
Residential home		66 (3.7)
Nursing home		74 (4.2)
Hospital		414 (23.3)
Palliative care unit/hospice		128 (7.2)
Other		28 (1.6)

Missing values: age 4, cause of death 31, dementia 17

### Provision of bereavement care

Table 2 shows the number of cases in which GPs had had contact or had plans to have contact with bereaved relatives of their deceased patient regarding bereavement care. In most cases GPs provided bereavement care or had plans to do so: 43.2% cases with contact once, 38.3% cases with contact multiple times and 4.9% cases in which the GP was planning to have contact. If a death was non-sudden it was more likely that the GP had provided bereavement care or had plans to do so (88.7%) than if a patient died suddenly (82.0%) (Odds ratio (OR) 1.60) (Table 3). The sensitivity analysis showed similar results, with a slightly lower OR (Additional file 2).

**Table 2:** Number of cases in which general practitioners had contact or had plans to have contact with bereaved relatives of their deceased patient regarding bereavement care, stratified by the suddenness of the death (*N* = 1779)

	Non-sudden death	Sudden death	Total *n* (%)
Yes, once	519 (44.6%)	249 (40.4%)	768 (43.2%)
Yes, multiple times	451 (38.8%)	230 (37.3%)	681 (38.3%)
No, but planned	62 (5.3%)	26 (4.2%)	88 (4.9%)
No, also not planned	131 (11.3%)	111 (18.0%)	242 (13.6%)

**Table 3:** Association between the provision of bereavement care by general practitioners and the death being expected and non-sudden (*n* = 1779)

	Contact related to bereavement care	
	Row %	OR (95% CI)
Sudden death (*n* = 616)	82.0%	1.00
Non-sudden death (*n* = 1163)	88.7%	1.60 (1.16–2.22)

*OR * odds ratio, *CI* confidence interval

Furthermore, within non-sudden deaths, several characteristics of deceased patients were associated with the provision of bereavement care by the patient’s general practitioner (Table 4). We found that in cases where an informal caregiver was involved in the last three months of life GPs were more likely to provide bereavement care (OR 3.81), and in cases where the GP was part of a palliative care at home group (PaTz group: groups of GPs and district nurses who meet bimonthly to identify and discuss their patients with support from a palliative care consultant) GPs were also more likely to provide bereavement care (OR 2.78). In cases where the patient died in a palliative care unit or hospice GPs were less likely to provide bereavement care (OR 0.53).

**Table 4:** Associations between the provision of bereavement care by a general practitioner and the characteristics of deceased patient who died non-suddenly (*n* = 1163)

		Contact related to bereavement care		
		**Row %**	GEE univariate OR (95% CI)	GEE multivariate OR (95% CI)
*Age (in years)*			1.00 (0.99–1.02)	
*Gender*				
Male (*n* = 633)		88.9%	1.00	
Female (*n* = 530)		88.5%	0.81 (0.55–1.20)	
*Main place of residence in the last year of life*				
Home/with family (*n* = 1100)		89.5%	1.00	1.00
Residential home (*n* = 63)		76.2%	**0.27 (0.14–0.53)**	0.39 (0.15-1.00)
*Cause of death*				
Cancer (*n* = 651)		88.3%	1.00	
Cardiovascular diseases (*n* = 122)		90.2%	1.05 (0.51–2.15)	
Respiratory diseases (*n* = 83)		86.7%	0.94 (0.50–1.78)	
Disorders of the nervous system (*n* = 31)		93.5%	1.09 (0.45–2.61)	
Stroke (CVA) (*n* = 28)		85.7%	0.97 (0.35–2.71)	
Old age (*n* = 137)		84.7%	0.79 (0.40–1.57)	
Other (*n* = 110)		95.5%	1.70 (0.82–3.54)	
*Dementia diagnosis*				
No (*n* = 997)		88.1%	1.00	
Yes (*n* = 157)		92.4%	1.98 (0.70–2.76)	
*Hospitalisation in the last month before death*				
No (*n* = 735)		87.9%	1.00	
Yes (*n* = 425)		90.1%	0.99 (0.72–1.37)	
*Emergency unit admission in the last month before death*				
No (*n* = 852)		87.8%	1.00	
Yes (*n* = 311)		91.3%	1.21 (0.86–1.70)	
Place of death*				
Home/with family (*n* = 759)		90.9%	1.00	1.00
Residential home (*n* = 46)		78.3%	**0.28 (0.13–0.60)**	0.62 (0.21–1.80)
Nursing home (*n* = 50)		84.0%	**0.40 (0.18–0.90)**	1.29 (0.33–5.11)
Hospital (*n* = 181)		88.4%	0.74 (0.44–1.25)	1.32 (0.49–3.55)
Palliative care unit/hospice (*n* = 123)		81.3%	**0.46 (0.22–0.95)**	**0.53 (0.28–0.99)**
*Year of death*				
2018 (*n* = 243)		88.9%	1.00	1.00
2019 (*n* = 223)		87.0%	0.74 (0.47–1.18)	0.69 (0.40–1.20)
2020 (*n* = 283)		85.5%	0.81 (0.45–1.45)	0.70 (0.33–1.49)
2021 (*n* = 289)		90.7%	1.48 (0.84–2.62)	1.38 (0.74–2.57)
2022 (*n* = 125)		94.4%	**2.01 (0.93–4.32)**	1.54 (0.78–3.06)
*Death at the preferred place*				
Yes (*n* = 886)		90.6%	1.00	
No (*n* = 94)		90.4%	0.76 (0.38–1.53)	
Don’t know (*n* = 181)		78.5%	0.68 (0.40–1.15)	
*Patient died peacefully*				
Yes (*n* = 143)		91.9%	1.00	
No (*n* = 1020)		66.4%	0.51 (0.19–1.35)	
*Patient could accept the imminent death*				
Yes, fully/mostly (*n* = 809)		91.7%	1.00	1.00
No, not fully/not at all (*n* = 170)		91.2%	1.04 (0.71–1.53)	1.14 (0.76–1.72)
Don’t know (*n* = 181)		72.9%	**0.58 (0.34–0.98)**	1.06 (0.48–2.34)
*GP provided palliative care to patient*				
Yes, until death (*n* = 829)		89.6%	1.00	1.00
Yes, but not until death (*n* = 74)		91.9%	0.98 (0.50–1.94)	1.58 (0.56–4.50)
No (*n* = 259)		84.9%	**0.62 (0.41–0.94)**	1.68 (0.81–3.51)
*Informal caregiver involved*				
No (*n* = 134)		61.9%	1.00	1.00
Yes (*n* = 1025)		92.2%	**4.57 (2.75–7.61)**	**3.81 (2.26–6.43)**
GP is part of a PaTz group**				
No (*n* = 830)		86.3%	1.00	1.00
Yes (*n* = 324)		94.8%	**2.24 (1.07–4.67)**	**2.78 (1.17–6.61)**

Missing values: cause of death 1, dementia 9, hospitalisation 1, place of death 4, death at the preferred place 2, patient could accept the imminent death 3, GP provided palliative care to patient 1, informal caregiver involved 3, GP is part of a PaTz group 9

*OR* odds ratio, *CI* confidence interval, *GP* general practitioner

*Place of death elsewhere not included because all relatives of patients who died elsewhere had had contact with the GP (100%) (outcome variable), so it was not possible to assess an association between this category and the outcome variable

** PaTz groups are groups of GPs and district nurses who meet bimonthly to identify and discuss their patients with support from a palliative care consultant

The sensitivity analyses showed similar associations between bereavement care and involvement of informal caregivers and GPs being part of a PaTz group (Additional file 2). Palliative care unit or hospice as the place of death was no longer associated with the provision of bereavement care, whereas admission to the emergency unit in the last 30 days before death was associated.

## Discussion

Our results indicate that in the vast majority of cases GPs in the Netherlands have contact with relatives of their deceased patients regarding bereavement care, and that this happens somewhat more often when patients die non-suddenly compared to unexpectedly and suddenly. Furthermore, when a patient’s death is non-sudden, GPs are more likely to provide relatives bereavement care if there is an informal caregiver involved in the care for their deceased patient, as well as when the GP is part of a PaTz group. On the other hand, GPs are less likely to provide bereavement care if the patient dies in a palliative care unit or hospice. No other case characteristics of deceased patients are associated with the provision of bereavement care by GPs.

The frequency of bereavement care following non-sudden deaths in our study (89%) is comparable to rates reported in studies conducted in other settings and time periods. For instance, a paper based on data from 2013-2014 from the same Sentinel network reported that GPs provided bereavement care in 93% of cases involving non-sudden deaths [15]. Furthermore, a study focusing on support for bereaved relatives following euthanasia or physician-assisted suicide showed that 82% of GPs had an conversation with relatives after euthanasia or physician-assisted suicide [16]. It is worth noting that the latter study only included data about conversations that had already taken place, whereas in our study also cases in which there were plans for future contact regarding bereavement care were included (5%).

In nearly 14% of the cases bereavement care was either not provided or not planned to be provided by the GP. Also when adding the cases that had not yet had contact but planned to do so (5%), this constitutes a small, but not negligible, number of cases in which bereavement care was not provided. Multiple reasons may account for a lack of bereavement care. Relatives may refuse the offer of their loved one’s GPs regarding bereavement care because they do not feel a need for bereavement care in general or prefer to discuss this with someone else. In certain cases the deceased patient may have had no relatives, but this is unknown based on this data. Furthermore, GPs may refrain from contacting bereaved relatives of their deceased patient due to time constraints or uncertainty regarding whether to routinely initiate contact or await the relatives’ consultation [1, 2]. Additionally, physicians might hesitate to offer bereavement care to all relatives out of concern that grief may be pathologized [1, 13]. Previous studies have demonstrated that many physicians lack formal education in grief and bereavement and physicians (and relatives) may thus benefit from training in bereavement care [1, 3, 17]. Finally, GPs might be hesitant to contact their patient’s bereaved relatives if these relatives are not their own patients.

Palliative care encompasses support not only for the patient but also for their relatives, with bereavement care being a core component [12]. Consequently, from a palliative care perspective, it is unjustifiable to refrain from providing bereavement care because the bereaved individual is not the GP’s own patient. The deceased patient’s GP may have information about the patient’s disease trajectory and death that is highly relevant for the bereavement process of the deceased patient’s relative [10, 16, 18, 19], implying that the deceased patient’s GP is in many cases well-positioned to provide this type of bereavement care. While the deceased patient’s GP may not be the designated physician for treating bereaved relatives with mental health issues in cases in which these are not the GP’s own patients, they can inquire about the relatives’ well-being and provide information on what to do if needed.

Multiple studies have advocated against a blanket approach for bereavement care (e.g. [20–22]), where every bereaved individual receives professional support, regardless of their risk for grief disorders or need. However, it seems like these studies often overlook the potential benefits of an initial professional bereavement care conversation, to identify bereaved individuals who need additional help. For example, it has been argued that offering specialized psychotherapeutic support to all bereaved individuals in palliative care, irrespective of need, is neither effective nor affordable [22, 23]. However, bereavement care does not necessarily have to be a comprehensive psychotherapeutic-oriented intervention. It can also be a one-time, low-threshold conversation with the GP. From a palliative care perspective, we argue that GPs have a responsibility to try to get in touch with bereaved relatives of their deceased patient. They can do this by checking the medical records for a registered contact person and reaching out regarding bereavement care. Given the frequency of bereavement care that we found, it appears that GPs in the Netherlands are already doing this frequently. If no contact person is registered in the deceased patient’s medical record and the GP is unaware of any close relatives, they will be unable to provide bereavement care. Improving education in grief and bereavement for physicians, emphasizing this responsibility, will likely increase the frequency of (low-threshold) bereavement care.

Previous research has shown that relatives of individuals who die suddenly are at an elevated risk of developing complicated grief [8]. In addition, Saunderson & Ridsdale found that GPs feel a particular responsibility to provide bereavement care in cases of sudden and unexpected deaths [24]. However, our study showed that GPs are more likely to provide bereavement care in cases where the individual’s death is non-sudden. This tendency may be explained by the higher likelihood of prior contact between the GP and the bereaved relatives in cases of non-sudden deaths compared to sudden deaths. Furthermore, in cases of sudden deaths GPs may find it more challenging to identify the bereaved relatives of their deceased patient, due to potentially limited prior contact compared to cases of non-sudden deaths [25]. Given the lack of evidence to clarify why bereavement care is less likely to be provided in cases of sudden death, we recommend further research into the reasons for this.

Among the group of individuals that die non-suddenly, we see that bereavement care by GPs is more common in cases involving an informal caregiver or when the GP was affiliated with a PaTz group. It is plausible that during the end-of-life phase, informal caregivers and GPs already interacted, such as during doctor’s appointments or home visits. This existing relationship potentially reduces the barrier for GPs to initiate contact regarding bereavement care [2]. Furthermore, in general, GPs participating in palliative care at home groups (PaTz) tend to have a stronger focus on palliative care and therefore may be more attentive to providing bereavement care compared to colleagues not in such groups. Previous research also showed that practices with an interest in palliative care are more likely to offer bereavement care [2]. On the contrary, if the patient died in a palliative care unit or hospice GPs were less likely to provide bereavement care compared to when a patient died at home. A possible explanation is that the palliative care responsibility, and thus the care for relatives, may lay with someone within this setting rather than with the GP. GPs may assume that palliative care units or hospices already pay much attention to bereavement care and that there is therefore not need for them to contact relatives with regards to bereavement care. Apart from involvement of informal caregivers, GPs being part of PaTz groups and dying in a palliative care unit or hospice, we found no other characteristics that were associated with the provision of bereavement care. This suggests that GPs’ decision to provide bereavement care may not be highly case-specific. This is encouraging, considering that bereaved relatives highly value GPs making time for them shortly after death [10].

### Strengths and limitations

This is one of the first studies to shed light on the proportion of cases in which GPs provide bereavement care and possible associations with case characteristics. In addition, the Nivel Sentinel Practices network covers approximately 0.8% of the Dutch population and is representative of the population for age, gender and population density. The sample may not be representative of other countries (e.g. different cause of death and place of death distribution), because we excluded deceased patients whose main place of residence in the last year of life was a nursing home. In the Netherlands, nursing home residents are no longer under the responsibility of a GP, which may differ in other countries. Moreover, GPs are mostly still involved in the care for patients who are admitted to a hospice or palliative care unit, and therefore responsible for their (bereaved relatives) which may also be different in other countries.

The use of data from the death registration questionnaire for the purposes of this study also had some limitations. As the death registration questionnaire focuses on the death of the patient based on physician-reported data, little information was available about the relatives of the deceased patient (e.g. Did the deceased patient have close relatives? Who were these close relatives? Were these relatives known to the GP and/or the GP’s patients?). We recommend that such data is collected in future studies on bereavement care, and that also the perspective of bereaved relatives regarding bereavement care provision by GPs of their deceased loved ones is further explored.

Another limitation of this study is the risk of social-desirability bias, meaning that GPs who have not yet contacted bereaved relatives might indicate plans to do so, even if they never actually will. We consider this risk relatively small, as fewer than 5% of GPs indicated plans bereavement care, compared to nearly 14% who indicated that they had no plans. The sensitivity analyses also showed little differences, when the “no, but planned” category was included in the “no bereavement care” category compare to when it was included in the “yes bereavement care” category. Also, based on this study we only know what share of GPs provide bereavement care and in what cases, but not what the bereavement care looks like. It would be interesting to investigate what the bereavement care that GPs report to provide actually entails. Finally, the study period (2018-2022) included the COVID-19 pandemic, which may have had an influence on bereavement care. Therefore, the year of death was included as a fixed effect in the model to correct for this. With this study, we did not gain insight into whether the content of bereavement care differed during the pandemic.

We recommend that future studies explore the perspective of bereaved relatives on the provision of bereavement care by the GP of their deceased loved one. This may include whether they received such care, their wishes and needs, and the content of the bereavement care received. Additionally, it would be valuable to gain more insight into the situations where the deceased and the bereaved relatives do not share the same GP, and to explore how these GPs (of the deceased patient and of the bereaved relative) view their responsibilities, task division and potential collaboration in providing bereavement care.

## Conclusion

Providing bereavement care seems to be common practice among GPs in the Netherlands. Our study demonstrated that in the majority of deaths, also with sudden death, GPs contact relatives of their patients regarding bereavement care. This is more prevalent when the patient died non-suddenly, when an informal caregiver was involved or when the GP was involved in a palliative care at home group (PaTz), and less prevalent when the patient died in a palliative care unit or hospice. The deceased person’s GP may have information about the end-of-life phase that is relevant for the bereavement process of the relatives. Given this and the responsibilities of physicians from a palliative care perspective, we feel that GPs should provide bereavement care to all relatives of their deceased patients, including relatives who are not their own patient. We believe it is important to raise awareness among GPs that their responsibility to care for bereaved relatives also applies in cases of sudden deaths of their patients. We recommend that they try to get into contact with these relatives, especially considering their elevated risk for grief disorders.

## Supplementary Information

Supplementary Material 1.

Supplementary Material 2.

## Data Availability

The dataset is available upon reasonable request by the corresponding author via eol@amsterdamumc.nl.

## Abbreviations

GP: general practitioner
GEE: generalized Estimating Equations
OR: odds ratio
CI: confidence interval

## References

[1] Ghesquiere AR, Patel SR, Kaplan DB, Bruce ML. Primary care providers’ bereavement care practices: recommendations for research directions. Int J Geriatr Psychiatry. 2014;29(12):1221–9. 24955568 10.1002/gps.4157 PMC4418789

[2] Nagraj S, Barclay S. Bereavement care in primary care: a systematic literature review and narrative synthesis. Br J Gen Pract. 2011;61(582):e42–8. 21401990 10.3399/bjgp11X549009 PMC3020071

[3] O’Connor M, Breen LJ. General practitioners’ experiences of bereavement care and their educational support needs: a qualitative study. BMC Med Educ. 2014;14(1):59. 24670040 10.1186/1472-6920-14-59 PMC3986890

[4] VGVZ [Association of Spiritual Caregivers]. Richtlijn rouw in de palliatieve fase [Guideline grief in the palliative phase]. 2022.

[5] Nielsen MK, Carlsen AH, Neergaard MA, Bidstrup PE, Guldin MB. Looking beyond the mean in grief trajectories: a prospective, population-based cohort study. Soc Sci Med. 2019;232:460–9. 31230666 10.1016/j.socscimed.2018.10.007

[6] Djelantik A, Smid GE, Mroz A, Kleber RJ, Boelen PA. The prevalence of prolonged grief disorder in bereaved individuals following unnatural losses: systematic review and meta regression analysis. J Affect Disord. 2020;265:146–56. 32090736 10.1016/j.jad.2020.01.034

[7] Lundorff M, Holmgren H, Zachariae R, Farver-Vestergaard I, O’Connor M. Prevalence of prolonged grief disorder in adult bereavement: a systematic review and meta-analysis. J Affect Disord. 2017;212:138–49. 28167398 10.1016/j.jad.2017.01.030

[8] Krychiw JK, James R, Ward-Ciesielski EF. Suddenness of death as a determinant of differential grief experiences. Bereave Care. 2018;37(3):92–100.

[9] Doering BK, Barke A, Vogel A, Comtesse H, Rosner R. Predictors of prolonged grief disorder in a German representative population sample: unexpectedness of bereavement contributes to grief severity and prolonged grief disorder. Front Psychiatry. 2022;13:853698. 35558417 10.3389/fpsyt.2022.853698 PMC9090313

[10] Van Goethem J, Verschaeve M, Peters S, Smeets M. Patients’ experiences and expectations of the General Practitioner’s role during bereavement care after losing a loved one: a qualitative study. Death Stud. 2023;47(6):751–61. 36063389 10.1080/07481187.2022.2119307

[11] Guldin M-B, Vedsted P, Jensen AB, Olesen F, Zachariae R. Bereavement care in general practice: a cluster-randomized clinical trial. Fam Pract. 2013;30(2):134–41. 22964078 10.1093/fampra/cms053

[12] Hudson P, Hall C, Boughey A, Roulston A. Bereavement support standards and bereavement care pathway for quality palliative care. Palliat Support Care. 2018;16(4):375–87. 28701233 10.1017/S1478951517000451

[13] Pearce C, Wong G, Kuhn I, Barclay S. Supporting bereavement and complicated grief in primary care: a realist review. BJGP Open. 2021;5(3):BJGPO.2021.0008. 33653707 10.3399/BJGPO.2021.0008 PMC8278512

[14] Veldhuijzen N, Hooiveld M, Overbeek L. Nivel Peilstations - voor aanvullende gegevens over ziekten [Nivel Sentinel Practices - for additional information about diseases] 2024 [updated May 9, 2024]. Available from: https://www.nivel.nl/nl/panels-en-registraties/nivel-zorgregistraties-eerste-lijn/methoden/nivel-peilstations. Cited 2024 May 23.

[15] Penders YWH, Onwuteaka-Philipsen B, Moreels S, Donker GA, Miccinesi G, Alonso TV, et al. Differences in primary palliative care between people with organ failure and people with cancer: an international mortality follow-back study using quality indicators. Palliat Med. 2018;32(9):1498–508. 30056802 10.1177/0269216318790386

[16] Renckens S, Pasman HR, van der Heide A, Onwuteaka-Philipsen B. Aftercare provision for bereaved relatives following euthanasia or physician-assisted suicide: a questionnaire study among physicians. Int J Public Health. 2024;69:1–8. 10.3389/ijph.2024.1607346 PMC11306013 39119216

[17] Atreya S, Datta SS, Salins N. Views of general practitioners on end-of-life care learning preferences: a systematic review. BMC Palliat Care. 2022;21(1):162. 36127706 10.1186/s12904-022-01053-9 PMC9490975

[18] Milberg A, Olsson E-C, Jakobsson M, Olsson M, Friedrichsen M. Family members’ perceived needs for bereavement follow-up. J Pain Symptom Manag. 2008;35(1):58–69. 10.1016/j.jpainsymman.2007.02.039 17949942

[19] Renckens SC, Onwuteaka-Philipsen BD, Jorna Z, Klop HT, du Perron C, van Zuylen L, et al. Experiences with and needs for aftercare following the death of a loved one in the ICU: a mixed-methods study among bereaved relatives. BMC Palliat Care. 2024;23(1):65. 38433194 10.1186/s12904-024-01396-5 PMC10910713

[20] Aoun SM, Rumbold B, Howting D, Bolleter A, Breen LJ. Bereavement support for family caregivers: the gap between guidelines and practice in palliative care. PLoS ONE. 2017;12(10):e0184750. 28977013 10.1371/journal.pone.0184750 PMC5627900

[21] Boven C, Dillen L, Van den Block L, Piers R, Van Den Noortgate N, Van Humbeeck L. In-hospital bereavement services as an act of care and a challenge: an integrative review. J Pain Symptom Manag. 2022;63(3):e295-316. 10.1016/j.jpainsymman.2021.10.008 34695567

[22] Breen LJ, Aoun SM, O’Connor M, Rumbold B. Bridging the gaps in palliative care bereavement support: an international perspective. Death Stud. 2014;38(1):54–61. 24521046 10.1080/07481187.2012.725451

[23] Aoun SM, Breen LJ, O’Connor M, Rumbold B, Nordstrom C. A public health approach to bereavement support services in palliative care. Aust N Z J Public Health. 2012;36(1):14–6. 22313700 10.1111/j.1753-6405.2012.00825.x

[24] Saunderson EM, Ridsdale L. General practitioners’ beliefs and attitudes about how to respond to death and bereavement: qualitative study. BMJ. 1999;319(7205):293–6. 10426743 10.1136/bmj.319.7205.293 PMC28183

[25] Stephen AI, Wilcock SE, Wimpenny P. Bereavement care for older people in healthcare settings: qualitative study of experiences. Int J Older People Nurs. 2013;8(4):279–89. 22309395 10.1111/j.1748-3743.2012.00319.x

