# Supplementary material for: The provision of bereavement care by general practitioners: data from a sentinel network
Source: BMC Prim Care. 2024 Oct 23;25:378. doi: 10.1186/s12875-024-02625-9 (PMC11515723; doi:10.1186/s12875-024-02625-9)
Supplement: Supplementary file 1 — Supplementary Material 1. [file 12875_2024_2625_MOESM1_ESM.docx]

**Additional file 1 – overview of variables**

| **A1. Overview of variables including recoding if applicable** | | |
| --- | --- | --- |
| **Variable** | **Original** | **Recoded** |
| *Contact between GPs and relatives related to bereavement care [dependent variable]* | | |
| Contact or plans to contact relatives with regards to bereavement care | 1. Yes, once 2. Yes, multiple times 3. No, but planned 4. No, and not planned | For the main analyses dichotomised into:   1. Yes (planned) (including yes, once; yes, multiple times; no but planned)­­^1^ 2. No, and not planned (including no, and not planned) |
| *Demographics [independent variables]* | | |
| Age | Continuous | NA |
|  |  |  |
| Gender | 1. Male 2. Female | NA |
|  |  |  |
| Main place of residence in the last year of life | 1. Home/with family 2. Residential home | NA |
|  |  |  |
| Cause of death | 1. Cancer 2. Cardiovascular diseases 3. Respiratory diseases 4. Disorders of the nervous system 5. Stroke (cardiovascular accident) 6. Old age 7. Other | NA |
|  |  |  |
| Dementia diagnosis | 1. Severe 2. Mild 3. None | 1. Yes 2. No |
| *Hospitalisation and emergency unit admission [independent variables]* | | |
| Hospital admission in the last 30 days before death | 1. None 2. Once 3. Two times or more | 1. No admissions 2. One or more admissions |
|  |  |  |
| Emergency unit admission in the last 30 days before death | 1. None 2. Once 3. Two times or more | 1. No admissions 2. One or more admissions |
| *Evaluation of the dying phase [independent variables]* | | |
| Sudden and completely unexpected death | 1. Yes 2. No | NA |
|  |  |  |
| Place of death | 1. Home/with family 2. Residential home 3. Nursing home 4. Hospital 5. Palliative care unit/ hospice 6. Elsewhere |  |
|  |  |  |
| Preferred place of death | 1. Yes 2. No 3. Don’t know |  |
|  |  |  |
| Death peaceful | 10- point Likert scale (1= no peaceful death, 10 = peaceful death) | 1. Did not die peacefully (score 1-5) 2. Died peacefully (score 6-10) |
|  |  |  |
| Acceptance of death by patient | 1. Yes, fully 2. Yes, mostly 3. No, not fully 4. No, not at all 5. Don’t know | 1. Yes, fully/mostly 2. No, not fully/ not at all 3. Don’t know |
| *Palliative care [independent variables]* | | |
| GP provided palliative care to patient | 1. Yes, until death 2. Yes, but not until death 3. No | NA |
|  |  |  |
| Part of a PaTz group (palliative care at home)^2^ | 1. Yes 2. No | NA |
| *Informal caregivers [independent variables]* | | |
| Most important informal caregiver in the last three months of life | 1. No informal caregiver 2. Cohabiting partner 3. Cohabiting son/ daughter 4. Other cohabiting informal caregiver 5. Non-cohabiting partner 6. Non-cohabiting son/ daughter 7. Other non-cohabiting informal caregiver | 1. Informal caregiver involved 2. No informal caregiver involved |
| 1. The answer category ‘no, but planned’ was included in the ‘yes’ category since in some cases the death registration questionnaire may have been completed shortly after death, leaving little time to already have had contact with bereaved relatives. 2. PaTz groups are groups of GPs and district nurses, supported by a palliative care consultant, who meet bimonthly to identify and discuss patients with palliative care needs (13). In 2023 there were 249 PaTz groups throughout the Netherlands (14). | | |
