# Supplementary material for: The provision of bereavement care by general practitioners: data from a sentinel network
Source: BMC Prim Care. 2024 Oct 23;25:378. doi: 10.1186/s12875-024-02625-9 (PMC11515723; doi:10.1186/s12875-024-02625-9)
Supplement: Supplementary file 2 — Supplementary Material 2. [file 12875_2024_2625_MOESM2_ESM.docx]

| **Table A1.** Association between the provision of bereavement care by general practitioners and the death being expected and non-sudden (N=1779) | | |
| --- | --- | --- |
|  | **Row %** | **OR (95% CI)** |
| Sudden death (n=616) | 77.8% | 1.00 |
| Non-sudden death (n=1163) | 83.4% | 1.39 (1.07-1.79) |
| OR = odds ratio; CI = confidence interval | | |

**Additional file 2 – sensitivity analyses**

| **Table A2.** Associations between the provision of bereavement care by a general practitioner and the characteristics of deceased patient who died non-suddenly (n=1163) | | | | |
| --- | --- | --- | --- | --- |
|  |  | **Contact related to bereavement counselling** | **GEE Univariate**  **OR (95% CI)** | **GEE multivariate**  **OR (95% CI)** |
| *Age (in years)* | |  | 1.01 (0.99-1.02) |  |
| *Gender* | |  |  |  |
|  | Male (n=633) | 84.2% | 1.00 |  |
|  | Female (n=530) | 82.5% | 0.80 (0.58-1.11) |  |
| *Main place of residence in the last year of life* | |  |  |  |
|  | Home/with family (n=1100) | 84.1% | 1.00 | 1.00 |
|  | Residential home (n=63) | 71.4% | **0.39 (0.20-0.77)** | 0.53 (0.22-1.27) |
| *Cause of death* | |  |  |  |
|  | Cancer (n=651) | 82.3% | 1.00 |  |
|  | Cardiovascular diseases (n=122) | 82.8% | 0.83 (0.49-1.41) |  |
|  | Respiratory diseases (n=83) | 85.5% | 1.34 (0.72-2.50) |  |
|  | Disorders of the nervous system (n=31) | 90.3% | 1.27 (0.54-3.02) |  |
|  | Stroke (CVA) (n=28) | 78.6% | 0.85 (0.30-2.39) |  |
|  | Old age (n=137) | 81.0% | 0.93 (0.53-1.66) |  |
|  | Other (n=110) | 90.9% | 1.46 (0.83-2.58) |  |
| *Dementia diagnosis* | |  |  |  |
|  | No (n=997) | 82.5% | 1.00 |  |
|  | Yes (n=157) | 88.5% | 1.47 (0.85-2.53) |  |
| *Hospitalisation in the last month before death* | |  |  |  |
|  | No (n=735) | 81.9% | 1.00 |  |
|  | Yes (n=425) | 85.9% | 1.15 (0.86-1.54) |  |
| *Emergency unit admission in the last month before death* | |  |  |  |
|  | No (n=852) | 81.8% | 1.00 | 1.00 |
|  | Yes (n=311) | 87.8% | **1.47 (1.07-2.02)** | **1.53 (1.11-2.12)** |
| *Place of death** | |  |  |  |
|  | Home/with family (n=759) | 85.0% | 1.00 | 1.00 |
|  | Residential home (n=46) | 76.1% | **0.50 (0.23-1.12)** | 0.95 (0.32-2.87) |
|  | Nursing home (n=50) | 80.0% | 0.58 (0.26-1.31) | 1.62 (0.52-5.05) |
|  | Hospital (n=181) | 85.1% | 0.98 (0.60-1.60) | 1.16 (0.61-2.21) |
|  | Palliative care unit/hospice (n=123) | 74.8% | 0.64 (0.36-1.13) | 0.69 (0.40-1.19) |
| *Year of death* | |  |  |  |
|  | 2018 (n=243) | 81.9% | 1.00 | 1.00 |
|  | 2019 (n=223) | 82.1% | 1.01 (0.65-1.59) | 0.98 (0.60-1.58) |
|  | 2020 (n=283) | 79.2% | 0.95 (0.59-1.54) | 0.90 (0.52-1.55) |
|  | 2021 (n=289) | 87.9% | **1.90 (1.08-3.36)** | **1.85 (1.05-3.26)** |
|  | 2022 (n=125) | 88.0% | 1.64 (0.70-3.85) | 1.49 (0.70-3.14) |
| *Death at the preferred place* | |  |  |  |
|  | Yes (n=886) | 85.0% | 1.00 |  |
|  | No (n=94) | 86.2% | 0.96 (0.56-1.63) |  |
|  | Don’t know (n=181) | 74.6% | 0.78 (0.54-1.13) |  |
| *Patient died peacefully* | |  |  |  |
|  | Yes (n=143) | 86.2% | 1.00 |  |
|  | No (n=1020) | 63.6% | 0.76 (0.33-1.79) |  |
| *Patient could accept the imminent death* | |  |  |  |
|  | Yes, fully/mostly (n=809) | 86.3% | 1.00 | 1.00 |
|  | No, not fully/not at all (n=170) | 85.9% | 1.09 (0.74-1.62) | 1.04 (0.67-1.60) |
|  | Don’t know (n=181) | 68.0% | **0.65 (0.41-1.03)** | 0.82 (0.41-1.63) |
| *GP provided palliative care to patient* | |  |  |  |
|  | Yes, until death (n=829) | 83.2% | 1.00 |  |
|  | Yes, but not until death (n=74) | 91.9% | 1.88 (0.85-4.13) |  |
|  | No (n=259) | 81.5% | 0.81 (0.58-1.14) |  |
| *Informal caregiver involved* | |  |  |  |
|  | No (n=134) | 54.6% | 1.00 | 1.00 |
|  | Yes (n=1025) | 87.1% | **4.02 (2.69-6.01)** | **3.90 (2.38-6.37)** |
| *GP is part of a PaTz group*** | |  |  |  |
|  | No (n=830) | 81.0% | 1.00 | 1.00 |
|  | Yes (n=324) | 89.5% | **1.95 (1.25-3.04)** | **2.07 (1.33-2.23)** |
| OR = odds ratio; CI = confidence interval; GP = general practitioner  *Place of death elsewhere not included because all had had contact with GP  ** PaTz groups are groups of GPs and district nurses who meet bimonthly to identify and discuss their patients with support from a palliative care consultant  Missing values: cause of death 1, dementia 9, hospitalisation 1, place of death 4, death at the preferred place 2, patient could accept the imminent death 3, GP provided palliative care to patient 1, informal caregiver involved 3, GP is part of a PaTz group 9 | | | | |
